# Long-term health effects of COVID-19 among patients in Croatian primary care settings

**DOI:** 10.3389/fmed.2026.1740432

**Published:** 2026-01-29

**Authors:** Branislava Popović, Ana Lesac Brizić, Aleksandar Ljubotina, Tina Zavidić, Vedrana Tudor Špalj, Roberta Marković Štimac, Martina Fišić Jurković, Nina Bašić Marković, Ines Diminić Lisica, Jasna Vučak, Nives Radošević Quadranti

**Affiliations:** 1 Department of Family Medicine, Faculty of Medicine, University of Rijeka, Rijeka, Croatia; 2 Family Medicine Practice Branislava Popović, Rijeka, Croatia; 3 Community Health Centre Primorsko Goranska County, Rijeka, Croatia; 4 Family Medicine Practice Aleksandar Ljubotina, Rijeka, Croatia; 5 Istrian Health Centres, Pazin, Croatia; 6 Primary Health Care Institution Srdoči, Rijeka, Croatia; 7 Health Institution dr. Ines Diminić Lisica, Rijeka, Croatia; 8 Family Medicine Practice Jasna Vučak, Sukošan, Croatia

**Keywords:** chronic diseases, COVID-19, long COVID, post-COVID syndrome, primary care, SARS-CoV-2

## Abstract

**Introduction:**

The COVID-19 pandemic has left lasting effects that extend beyond the acute phase of infection, with increasing evidence of long-term health consequences. This study aimed to assess the prevalence of post-COVID symptoms and conditions and to identify associated risk factors, including pre-existing chronic diseases, COVID-19 vaccination status, and severity of acute infection.

**Methods:**

This retrospective cross-sectional study was conducted in 10 family medicine practices in Croatia. The data collected from medical records included demographics, COVID-19 vaccination status, SARS-CoV-2 infection history and severity, and documented health conditions before and after infection. Descriptive statistics were used to summarize the data. Group differences were analyzed using the independent samples *t*-test or χ^2^ test. Variables significant in univariate analyses (*p* < 0.05) were included into multivariate regression models. Multiple linear regression was used to identify predictors of COVID-19 severity, and binary logistic regression was applied to determine factors associated with post-COVID conditions. Results are presented as regression coefficients (*β*) or odds ratios (OR) with 95% confidence intervals (CI). A *p*-value < 0.05 was considered statistically significant.

**Results:**

The study included 1,423 participants (58.0% female; mean age 52.6 ± 17.2 years), of whom 82.4% had confirmed SARS-CoV-2 infection and 32.3% were unvaccinated. At least one chronic disease was present in 28.1% of participants. The most frequently reported post-COVID conditions were brain fog (4.9%), neurological disorders (4.7%), cardiovascular diseases (2.9%), shortness of breath (2.8%), obesity (2.7%) and mental health disorders (2.6%). Greater COVID-19 severity was independently associated with pulmonary disease (*β* = 0.22; *p* = 0.031) and older age, particularly 51–65 years (*β* = 0.31; *p* < 0.001) and ≥66 years (*β* = 0.50; *p* < 0.001). COVID-19 vaccination was associated with milder disease (*β* = −0.21; *p* < 0.001). Previous cardiovascular and musculoskeletal diseases significantly increased the risk of thromboembolism. Diabetes, obesity, and number of vaccine doses were predictors of brain fog, while neurological comorbidities predicted post-COVID mental health disorders.

**Conclusion:**

Post-COVID symptoms and conditions represent an important long-term public health challenge. Family medicine physicians play a key role in early recognition, monitoring, and management of post-COVID sequelae, contributing to improved long-term patient outcomes.

## 1 Introduction

The COVID-19 pandemic, caused by the SARS-CoV-2 virus, has posed a significant challenge to healthcare systems worldwide. A growing body of evidence indicates that the impact of SARS-CoV-2 infection does not necessarily end with viral clearance. Numerous studies have demonstrated that SARS-CoV-2 infection can lead to long-term health consequences that persist well beyond the acute phase of disease (1).

The World Health Organisation (WHO) developed a clinical case definition for post-COVID-19 condition—commonly known as “long COVID”—using a Delphi consensus process involving global experts. Post-COVID-19 condition occurs in individuals with a history of probable or confirmed SARS-CoV-2 infection, usually 3 months from the onset of COVID-19, with symptoms that last for at least 2 months and cannot be explained by an alternative diagnosis (2). The NASEM Committee recently updated the definition of long COVID, emphasizing three key changes to improve understanding and recognition. The definition explains that the phrase “long COVID” is widely recognized and easy for both the public and healthcare professionals to understand. Long COVID is now considered part of a broader category of illnesses that can occur after infections (3). However, Croatia does not have a national long COVID clinical guideline for healthcare professionals with a standardized diagnostic protocol. Depending on the affected organ system and the severity of symptoms, GPs refer patients to a neurologist, cardiologist, etc. The Croatian healthcare system has developed a set of national recommendations and educational materials addressing post-COVID-19 condition, primarily coordinated by the Croatian Institute of Public Health (4). These documents aim to support both healthcare professionals and patients in the recognition, assessment, and management of persistent symptoms after SARS-CoV-2 infection.

Outpatient clinics for the care of patients with post-COVID symptoms have been established in several Croatian hospitals, including a pediatric outpatient clinic, as well as a post-COVID day hospital for physical and rehabilitation medicine. However, the absence of fully standardized national clinical pathways highlights the ongoing need for further systematization, research, and evaluation of post-COVID-19 care models.

The clinical course and outcomes of SARS-CoV-2 infection vary widely among individuals, ranging from asymptomatic to severe disease requiring hospitalization and intensive care. Earlier research has identified multiple risk factors for severe forms of SARS-CoV-2 infection, particularly chronic diseases (5). Previous studies have examined the associations between SARS-CoV-2 infection and incidence of newly developed chronic conditions, such as diabetes mellitus, cardiovascular, neurological and psychiatric diseases (6–9).

The introduction of vaccination against SARS-CoV-2 infection played a key role in controlling the pandemic, but vaccination acceptance was heterogeneous. COVID-19 vaccination behavior depended on several individual and health characteristics, including the presence of comorbidities. Apart from the severity of symptoms, the consequences of the SARS-CoV-2 infection can also vary (10). Despite extensive research on vaccine efficacy and determinants of severe disease, less is known about how chronic diseases before and after SARS-CoV-2 infection affect two key outcomes: the number of vaccine doses received and the severity of SARS-CoV-2 infection.

Epidemiological data indicate that both *brain fog* and mental health disorders affect approximately one in five individuals within 3 months to 2 years post-infection, underscoring a significant public health concern (11). Frequent symptoms include fatigue, shortness of breath, and cognitive dysfunction (often referred to as *brain fog*), which generally have an impact on everyday functioning. Symptoms may be new-onset following initial recovery from an acute COVID-19 episode or persist from the initial illness (12, 13).

Mental health conditions included diagnosed mental illness as well as symptoms such as anxiety or low mood, associated with distress or impairment in functioning or future incidence of full disorder without reaching the full criteria for a mental disorder at that time (11).

This study aimed to assess the prevalence and risk factors for the development of post-COVID symptoms and diseases in the general population, taking into account pre-existing chronic diseases and the severity of the clinical picture of SARS-CoV-2 infection. Particular attention was given to the occurrence of *brain fog* and shortness of breath and their associations with disease severity, vaccination status and comorbidities.

## 2 Methods

### 2.1 Data collection

This retrospective cross-sectional study was conducted in Croatia. The study included 10 family medicine practices selected through systematic convenience sampling. The study was not designed to achieve national representativeness. A systematic sample was used, with every tenth patient from the list (in alphabetical order) included in the analysis. The data collected from medical records included patients’ gender, age, COVID-19 vaccination status, history and severity of SARS-CoV-2 infection/s, and documented diseases and symptoms/disorders occurring before and after the SARS-CoV-2 infection.

Since the COVID-19 pandemic was officially declared in Croatia in March 2020, data on infection rates and disease severity were systematically tracked until the pandemic formally concluded in May 2023.

Chronic comorbidities were grouped by organ systems. Arterial hypertension, diabetes and obesity were observed separately. Post-COVID outcomes included self-reported new-onset symptoms such as neurological disorders (headache, persisting smell/taste abnormalities, slowness of movement, peripheral neuropathy, sleep disturbances, tinnitus), *brain fog*, shortness of breath, and mental health conditions. A cutoff point of 12 weeks was used to extract and analyze the relevant data after acute SARS-CoV-2 infection.

The severity of the SARS-CoV-2 infection was determined based on the clinical assessment from the medical documentation. Disease severity was assessed according to clinical criteria outlined in the Croatian Ministry of Health’s Guidelines for the Treatment of Patients with COVID-19, Version 2, dated November 19, 2020 (14). Based on the symptoms of the disease, the patients were divided into 6 groups: asymptomatic, mild (various symptoms, fever, general malaise, headache, myalgia, runny nose, sore throat and/or cough), moderate (severe symptoms of the disease and/or pneumonia, without the need for oxygen replacement therapy – SpO2 > 93% on room air), severe (respiratory frequency >30 breaths/min, respiratory failure or the need for oxygen replacement therapy – SpO2 ≤ 93% on room air), critical (criteria for ARDS, sepsis, septic shock, with/without acute dysfunction of other organ systems-shock, renal failure, coagulopathy, impaired consciousness) and patients with a fatal outcome.

### 2.2 Data analysis

The normality of the distribution of numerical variables was tested using the Kolmogorov–Smirnov test, which did not show a significant deviation from normality (*p* > 0.05). Therefore, parametric statistical methods were used, and values were presented as means ± standard deviations (SD) for continuous variables and as absolute and frequencies (n, %) for categorical variables.

In univariate analyses, the independent samples *t*-test and χ^2^ test of independence were applied, depending on the type of variable. Variables that showed statistical significance (*p* < 0.05) were included in the multivariate logistic regression analysis. Multiple linear regression analysis was used to identify predictors of SARS-CoV-2 infection severity, while binary logistic regression was applied to examine factors associated with post-COVID conditions.

COVID-19 vaccination was operationalized differently across regression models depending on the outcome of interest. For analyses of post-COVID clinical conditions, vaccination status was included as a binary variable (vaccinated vs. unvaccinated), reflecting clinically relevant exposure. For analyses of neurocognitive and subjective post-COVID symptoms, such as brain fog, the number of COVID-19 vaccine doses was included as a continuous variable to explore potential dose–response associations.

Age and sex were included as covariates in all binary logistic regression models. Variables that were not statistically significant (*p* > 0.05) and whose exclusion did not change the remaining coefficients by more than 10% were removed, and parsimonious models including clinically relevant predictors were presented.

Analyses were performed in the IBM SPSS Statistics version 24.0 and Stata version 15.0. The results of the logistic regression are presented as odds ratios (OR) with the corresponding 95% confidence intervals (CI), and *p* values < 0.05 were considered statistically significant.

## 3 Results

The study was conducted in 10 family medicine practices, including five located in urban areas and five in rural areas. Table 1 presents the characteristics of the physicians who participated in the survey.

**Table 1:** Characteristics of 10 participating GP offices.

Urban/rural setting	Number of patients	Average patients’ age	GP sex	GP years of age	GP years of experience
Rural	2,197	45.3	Female	39	15
Rural	2,010	49.7	Female	52	23
Rural	1,354	55.1	Female	44	18
Rural	1,728	62.2	Female	65	41
Rural	2,120	50	Female	57	31
Urban	2,194	49.3	Female	59	32
Urban	1,557	65.7	Male	68	43
Urban	1,723	55	Female	48	13
Urban	1,701	60.1	Female	66	41
Urban	1,996	44.2	Female	34	10

### 3.1 Study population characteristics

The study included 1,423 subjects, of whom 58.0% were female. The mean age of the participants was 52.6 ± 17.2 years (range 9–100). The largest number of subjects belonged to the 36–50-year age group (34.3%), while the smallest number was younger than 35 years (14.7%). Of the total number of subjects, 82.4% had a confirmed SARS-CoV-2 infection. Almost two-thirds of those infected described the course of the disease as mild (65.5%), while a moderate-to-severe form of the disease (≥3 according to the severity scale) was recorded in 10.6% of cases. An asymptomatic course was observed in 8.2% of subjects, and a critical form of the disease was recorded in 0.5% of cases. Regarding vaccination against COVID-19, 32.3% of respondents did not receive any dose of the vaccine, and 38.2% of respondents had two doses. Twenty percent of respondents received three doses, while 1.8% received four or more doses (Table 2).

**Table 2:** Sociodemographic characteristics of participants and clinical characteristics related to COVID-19 (*N* = 1,423).

Variable	Category	n	% (M ± SD)
Sex	Female	825	58.0
	Male	598	42.0
Age (years)	52.6 ± 17.2 (range: 9–100)		
Age groups	≤35	209	14.7
	36–50	488	34.3
	51–65	357	25.1
	≥66	369	25.9
COVID-19 vaccination	0 doses	459	32.3
	1 dose	106	7.4
	2 doses	544	38.2
	3 doses	289	20.3
	≥4 doses	25	1.8
History of COVID-19	Yes	1,172	82.4
	No	251	17.6
Number of infections (*N* = 1,423)	0 (never infected)	251	17.6
	1	688	48.3
	2	297	20.9
	3	48	3.4
	4	139	9.8
COVID-19 severity (*N* = 1,172)	1 - Asymptomatic	116	8.2
	2 - Mild	932	65.5
	3 – Moderate	76	5.3
	4 - Severe	24	1.7
	5 -Critical	7	0.5
	6 -Death	17	1.2
COVID-19 severity ≥3 (*N* = 1,172)		124	10.6

df, degree of freedom; SD, standard deviation; M = mean.

### 3.2 Pre-existing comorbidities and newly diagnosed conditions after SARS-CoV-2 infection

Before the SARS-CoV-2 infection, 71.9% of respondents had no chronic condition, while 28.1% had at least one chronic disease. The most frequently reported comorbidities were arterial hypertension (24.9%), cardiovascular diseases (12.8%), and thyroid disorders (6.2%). After recovering from the infection, the most frequently reported post-COVID symptoms were *brain fog* (4.9%) and shortness of breath (2.8%). Of the newly diagnosed diseases after infection, the most common were neurological (4.7%) and cardiovascular (2.9%) diseases, followed by obesity (2.7%) and mental health disorders (2.6%) (Tables 3, 4).

**Table 3:** Chronic diseases before and after SARS-CoV-2 infection (N = 1,423).

**Diseases**	**Before**		**After**	
	***N***	**%**	***N***	**%**
Arterial hypertension	355	24.9	32	2.2
Cardiovascular diseases (except arterial hypertension)	182	12.8	37	2.9
Pulmonary disease	60	4.2	18	1.3
Gastrointestinal	76	5.4	7	0.5
Thyroid diseases	74	6.2	17	1.2
Musculoskeletal diseases	71	5.0	24	1.9
Kidney disease	17	1.2	2	0.2
Malignant disease	76	5.3	15	1.1
Diabetes mellitus	58	4.1	13	0.9
Neurological disorders	32	2.2	67	4.7
Obesity	58	4.1	38	2.7
Mental health	81	5.7	37	2.6

**Table 4:** New post-COVID-19 symptoms and diagnosis in the study population (*N* = 1,423).

**New post-COVID-19 symptom**	***n***	**%**
*Brain fog*	70	4.9
Shortness of breath	40	28
Neurological disorders	67	4.7
Mental health disorders	37	2.6
Obesity	38	2.7
Diabetes	13	0.9
Pulmonary thromboembolism	14	1.0

### 3.3 Vaccination status and comorbidity patterns

Chronic diseases were more common in multiply vaccinated subjects, especially in those with three or more doses (over 40%) (Table 5). The χ^2^ test of independence showed a significant association between the number of vaccinations and the presence of chronic diseases [χ^2^(6, *N* = 1,172) = 31.01, *p* < 0.001, Cramer’s *V* = 0.163], and as the number of vaccinations increases, the proportion of subjects with at least one chronic disease also increases. Specifically, while 30.3% of unvaccinated subjects had a chronic disease, among those with three or more doses, this proportion increases to more than 40%.

**Table 5:** Association between COVID-19 vaccination status and presence of chronic diseases (*N* = 1,172).

Number of vaccine doses	No chronic disease		≥1 chronic disease (n)		Total
	*n*	%	*n*	%	
0 doses	272	69.7	118	30.3	390
1 dose	65	77.4	19	22.6	84
2 doses	335	75.1	111	24.9	446
≥3 doses	145	57.5	107	42.5	252
Total	817	69.7	355	30.3	1,172

*χ*^2^(6, *N* = 1,172) = 31.01, *p* < 0.001; Cramer’s *V* = 0.163.

The highest vaccination rates were observed among patients with malignant diseases (79.7%) and diabetes (79.3%), followed by chronic kidney disease patients (76.5%) and chronic respiratory diseases (73.3%).

### 3.4 Factors associated with COVID-19 severity

The results showed that pulmonary disease was a significant positive predictor of disease severity (*β* = 0.22, 95% CI [0.02, 0.42], *p* = 0.031). Compared with the reference group under 35 years of age, people aged 51–65 years (*β* = 0.31, 95% CI [0.17, 0.44], *p* < 0.001) and 66 years and older (*β* = 0.50, 95% CI [0.37, 0.64], *p* < 0.001) had significantly more severe COVID-19, while the group 36–50 years showed a trend toward more severe disease (*β* = 0.13, *p* = 0.049).

Vaccination was a significant negative predictor of disease severity (*β* = −0.21, 95% CI [−0.30, −0.12], *p* < 0.001), indicating that vaccinated individuals had a milder form of COVID-19 (Table 6).

**Table 6:** Multiple linear regression results for predictors of COVID-19 severity.

Predictor	*B*	95% CI	*p*
(Constant)	1.965	[1.857, 2.073]	<0.001
Pulmonary disease (yes)	0.219	[0.020, 0.417]	0.031
Age 36–50 years	0.127	[0.001, 0.253]	0.049
Age 51–65 years	0.310	[0.174, 0.445]	<0.001
Age ≥66 years	0.504	[0.368, 0.642]	<0.001
Vaccinated (yes)	−0.207	[−0.297, −0.118]	<0.001
Model fit:	*F*(5, 1,166) = 17.58, *p* < 0.001, *R*^2^ = 0.070, Adj. *R*^2^ = 0.067.		

### 3.5 Factors associated with post-COVID cardiovascular, musculoskeletal, pulmonary thromboembolism, and pulmonary diseases

Binary logistic regression analysis was performed to examine factors associated with the occurrence of cardiovascular disease, musculoskeletal disease, pulmonary thromboembolism, and pulmonary disease after SARS-CoV-2 infection.

#### 3.5.1 Cardiovascular disease

Individuals with at least one chronic disease were more likely to develop cardiovascular complications (OR = 3.17; 95% CI 1.27–7.87; *p* = 0.013). Musculoskeletal disease before SARS-CoV-2 infection was also associated with an increased risk (OR = 3.28; 95% CI 1.29–8.32; *p* = 0.012), as was arterial hypertension (OR = 2.70; 95% CI 1.12–6.50; *p* = 0.027).

#### 3.5.2 Musculoskeletal disorders

The presence of at least one chronic disease was associated with a higher likelihood of developing musculoskeletal disorders after SARS-CoV-2 infection (OR = 2.18; 95% CI 0.95–5.01; *p* = 0.067). COVID-19 vaccination showed a significant protective effect, as vaccinated individuals were less likely to develop musculoskeletal disorders in the post-COVID period (OR = 0.26; 95% CI 0.11–0.61; *p* = 0.002).

#### 3.5.3 Pulmonary thromboembolism

Moderate-to-severe COVID-19 was strongly associated with an increased risk of pulmonary thromboembolism compared with milder forms of the disease (OR = 69.25; 95% CI 13.33–359.65; *p* < 0.001). Previous cardiovascular disease before COVID-19 (OR = 5.96; 95% CI 1.03–34.39; *p* = 0.046) and musculoskeletal disease before COVID-19 (OR = 7.78; 95% CI 1.03–58.67; *p* = 0.047) were significant predictors. In contrast, arterial hypertension before COVID-19 was associated with a lower risk of pulmonary thromboembolism (OR = 0.13; 95% CI 0.02–0.93; *p* = 0.042). COVID-19 vaccination showed a non-significant trend toward a protective effect (OR = 0.31; 95% CI 0.09–1.09; *p* = 0.067).

#### 3.5.4 Pulmonary disease

Severe COVID-19 was associated with an increased likelihood of developing pulmonary disease after infection (OR = 2.98; 95% CI 1.05–8.45; *p* = 0.040). Arterial hypertension before COVID-19 was also a significant predictor (OR = 4.70; 95% CI 1.72–12.80; *p* = 0.002). Although statistical significance was not reached, COVID-19 vaccination showed a trend toward a reduced risk of pulmonary disease (OR = 0.42; 95% CI 0.16–1.09; *p* = 0.074) (Table 7).

**Table 7:** Logistic regression models for factors associated with post-COVID cardiovascular, musculoskeletal, pulmonary thromboembolism and pulmonary diseases.

Variable	Cardiovascular diseases OR (95% CI)	*p*	Musculoskeletal diseases OR (95% CI)	*p*	Pulmonary thromboembolism OR (95% CI)	*p*	Pulmonary diseases OR (95% CI)	*p*
Sex (male)	0.55 (0.23–1.28)	0.164	1.20 (0.52–2.79)	0.665	3.65 (0.99–13.47)	0.052*	0.48 (0.17–1.39)	0.178
Age	—	—	—	—	—	—	—	—
≥1 chronic disease	3.17 (1.27–7.87)	0.013**	2.18 (0.95–5.01)	0.067*	—	—	—	—
Severe COVID-19	—	—	—	—	69.25 (13.33–359.65)	<0.001***	2.98 (1.05–8.45)	0.040**
Cardiovascular diseases (before COVID-19)	—	—	—	—	5.96 (1.03–34.39)	0.046**	—	—
Musculoskeletal diseases (before COVID-19)	3.28 (1.29–8.32)	0.012**	—	—	7.78 (1.03–58.67)	0.047**	—	—
Pulmonary diseases (before COVID-19)	—	—	—	—	3.51 (0.76–16.06)	0.106	—	—
Arterial hypertension	2.70 (1.12–6.50)	0.027**	—	—	0.13 (0.02–0.93)	0.042**	4.70 (1.72–12.80)	0.002***
Obesity	—	—	—	—	—	—	—	—
COVID-19 vaccination	—	—	0.26 (0.11–0.61)	0.002***	0.31 (0.09–1.09)	0.067*	0.42 (0.16–1.09)	0.074*
Model fit	LR χ^2^(4) = 38.39; *p* < 0.001; Pseudo *R*^2^ = 0.134; *n* = 1,172		LR χ^2^(3) = 13.36; *p* = 0.004; Pseudo *R*^2^ = 0.059; *n* = 1,172		LR χ^2^(6) = 54.37; *p* < 0.001; Pseudo *R*^2^ = 0.381; *n* = 1,172		LR χ^2^(4) = 19.24; *p* < 0.001; Pseudo *R*^2^ = 0.103; *n* = 1,172	

*p < 0.10, **p < 0.05, ***p < 0.01.

### 3.6 Factors associated with post-COVID gastrointestinal, endocrine, and cardiovascular conditions

Binary logistic regression analysis was performed to examine factors associated with the occurrence of gastrointestinal diseases, thyroid diseases, arterial hypertension and diabetes after SARS-CoV-2 infection.

#### 3.6.1 Gastrointestinal diseases

The analysis showed that severe COVID-19 (*p* = 0.005) and previous cardiovascular disease (*p* = 0.001) significantly increased the risk of developing gastrointestinal diseases after infection. Severe COVID-19 was associated with a higher likelihood of developing gastrointestinal diseases after infection (OR = 12.45; 95% CI 2.11–73.41; p = 0.005).

#### 3.6.2 Thyroid diseases

Men were less likely than women to develop thyroid disease after COVID-19 (OR = 0.31, 95% CI [0.09–1.09], *p* = 0.068). Vaccination also showed a protective trend (OR = 0.42, 95% CI [0.16–1.10], *p* = 0.079). Although no predictor reached statistical significance, the observed trends suggest a higher propensity for post-COVID thyroid dysfunction in women and a potential protective effect of vaccination.

#### 3.6.3 Cardiovascular diseases and arterial hypertension

Individuals with cardiovascular disease before COVID-19 had a markedly increased risk (OR = 30.04; 95% CI 4.31–209.38; *p* = 0.001). Among the predictors analyzed, obesity was a significant risk factor (OR = 3.27, 95% CI [1.10–9.76], *p* = 0.034).

#### 3.6.4 Obesity and diabetes

Obesity before COVID-19 showed a trend toward increased gastrointestinal risk (OR = 8.31; *p* = 0.080), whereas arterial hypertension before COVID-19 was protective (OR = 0.04; *p* = 0.022). Pulmonary diseases were associated with a higher, but not statistically significant, risk (OR = 3.51, 95% CI [0.76–16.06], *p* = 0.106). Individuals who had musculoskeletal diseases before infection were significantly more likely to develop diabetes after COVID (OR = 5.60, 95% CI [1.49–21.02], *p* = 0.011) (Table 8).

**Table 8:** Logistic regression models for factors associated with post-COVID gastrointestinal diseases, thyroid diseases, arterial hypertension, and diabetes.

Variable	Gastrointestinal diseases OR(95% CI)	*p*	Thyroid diseases OR (95% CI)	*p*	Arterial hypertension (after COVID-19) OR (95% CI)	*p*	Diabetes (after COVID-19) OR (95% CI)	*p*
Sex (male)	—	—	0.31 (0.09–1.09)	0.068*	—	—	—	—
Age	—	—	—	—	1.01 (0.99–1.03)	0.315	—	—
≥1 chronic disease	—	—	—	—	—	—	—	—
Severe COVID-19	12.45 (2.11–73.41)	0.005***	2.98 (1.05–8.45)	0.040**	—	—	—	—
Cardiovascular diseases (before COVID-19)	30.04 (4.31–209.38)	0.001***	—	—	0.40 (0.09–1.85)	0.243	—	—
Musculoskeletal diseases (before COVID-19)	—	—	—	—	—	—	5.60 (1.49–21.02)	0.011**
Pulmonary diseases (before COVID-19)	—	—	3.51 (0.76–16.06)	0.106	—	—	—	—
Arterial hypertension	0.04 (0.00–0.62)	0.022**	—	—	—	—	—	—
Obesity	8.31 (0.78–88.92)	0.080*	—	—	3.27 (1.10–9.76)	0.034**	1.94 (0.24–15.56)	0.534
COVID-19 vaccination	—	—	0.42 (0.16–1.10)	0.079*	—	—	0.41 (0.14–1.23)	0.112
Model fit	LR *χ*^2^(4) = 19.24; *p* < 0.001; Pseudo *R*^2^ = 0.243; *n* = 1,172		LR χ^2^(3) = 8.75; *p* = 0.033; Pseudo *R*^2^ = 0.049; *n* = 1,172		LR χ^2^(3) = 5.33; *p* = 0.149; Pseudo *R*^2^ = 0.018; *n* = 1,172		LR χ^2^(3) = 7.45; *p* = 0.059; Pseudo *R*^2^ = 0.052; *n* = 1,172	

*p < 0.10, **p < 0.05, ***p < 0.01.

### 3.7 Factors associated with brain fog, psychiatric disorders, and dyspnea after COVID-19

#### 3.7.1 Brain fog

In the logistic regression model for the occurrence of *brain fog*, the number of doses of the COVID-19 vaccine received, the presence of diabetes and obesity were significant predictors. Respondents who received a higher number of vaccine doses were 1.84 times more likely (OR = 1.839; 95% CI: 1.44–2.36; *p* < 0.001) to develop *brain fog* compared to those who received fewer doses. People with diabetes were three times more likely to develop *brain fog* (OR = 3.03; 95% CI: 1.07–8.62; *p* = 0.037), while obesity was also a significant predictor (OR = 2.59; 95% CI: 1.03–6.52; *p* = 0.043) - obese respondents were about 2.6 times more likely to develop *brain fog*. Men showed about 40% lower probability of *brain fog* compared to women, but with a low level of significance (Table 9).

**Table 9:** Logistic regression models for factors associated with *brain fog*, psychiatric disorders, and dyspnea after COVID-19.

Variable	Brain fog OR (95% CI)	*p*	Psychiatric disorders OR (95% CI)	*p*	Dyspnea OR (95% CI)	*p*
Sex (male)	0.600 (0.34–1.05)	0.073*	1.578 (0.77–3.23)	0.211	0.718 (0.35–1.48)	0.370
Number of COVID-19 vaccinations	1.839 (1.44–2.36)	<0.001***	—	—	—	—
Number of COVID-19 infections	—	—	1.311 (0.97–1.78)	0.082*	0.958 (0.66–1.39)	0.819
≥1 chronic disease	1.096 (0.77–1.56)	0.610	—	—	0.056 (0.01–0.24)	<0.001***
Cardiovascular diseases	1.305 (0.55–3.10)	0.546	—	—	—	—
Thyroid diseases	0.500 (0.12–2.15)	0.352	—	—	—	—
Diabetes	3.034 (1.07–8.62)	0.037**	1.798 (0.49–6.62)	0.378	—	—
Neurological disorders	0.701 (0.09–5.55)	0.736	4.106 (1.08–15.56)	0.038*	—	—
Obesity	2.593 (1.03–6.52)	0.043**	—	—	2.463 (0.69–8.86)	0.167
Severe COVID-19 (≥3)	—	—	—	—	4.876 (2.18–10.89)	<0.001***
Pulmonary diseases	—	—	—	—	13.248 (4.43–39.60)	<0.001***
Model fit	LR χ^2^(8) = 39.60; *p* < 0.001; Pseudo *R*^2^ = 0.077; *n* = 1,172		LR χ^2^(4) = 9.82; *p* = 0.043; Pseudo *R*^2^ = 0.033; *n* = 1,172		LR χ^2^(6) = 45.30; *p* < 0.001; Pseudo *R*^2^ = 0.139; *n* = 1,172	

*p < 0.10, **p < 0.05, ***p < 0.001. OR = odds ratio; CI = confidence interval.

#### 3.7.2 Mental health disorders

In the model for the occurrence of mental health disorders after SARS-CoV-2 infection, the presence of neurological disorders was shown to be a significant predictor; respondents were approximately four times more likely to develop a mental health disorder compared to those without neurological comorbidities (OR = 4.11; 95% CI: 1.08–15.56; *p* = 0.038). The number of SARS-CoV-2 infections showed a borderline significant association (*p* = 0.082), with each additional infection increasing the likelihood of mental health disorder by approximately 31% (OR = 1.31; 95% CI: 0.97–1.78) (Table 9).

#### 3.7.3 Shortness of breath

The most important predictors for the occurrence of shortness of breath after recovering from SARS-CoV-2 infection were the severity of the acute infection, the presence of chronic pulmonary disease and the status of comorbidities. Subjects who had a moderate or severe form of the disease (severity ≥ 3) were almost five times more likely to have shortness of breath (OR = 4.86; 95% CI: 2.20–10.72; *p* < 0.001). Pulmonary disease was the strongest predictor, whereby subjects with this diagnosis had as much as 13 times greater odds of developing shortness of breath (OR = 13.78; 95% CI: 4.62–41.09; p < 0.001).

On the other hand, the presence of at least one chronic disease (other than pulmonary) was shown to be a protective factor, as such subjects were approximately 95% less likely to develop shortness of breath compared to subjects without comorbidities (OR = 0.053; 95% CI: 0.012–0.226; *p* < 0.001). Obesity was not a significant predictor (*p* = 0.187), although obese subjects were approximately 2.4 times more likely to develop dyspnea (Table 9).

**Figure 1:**
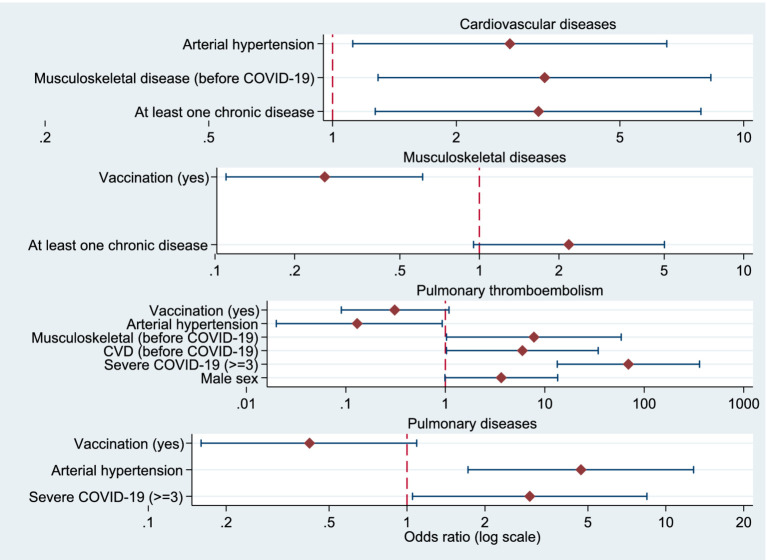
Forest plots showing odds ratios (OR) and 95% confidence intervals for factors associated with post-COVID cardiovascular diseases, musculoskeletal diseases, pulmonary thromboembolism, and pulmonary diseases. The vertical dashed line indicates OR = 1. All *x*-axes are presented on a logarithmic scale.

**Figure 2:**
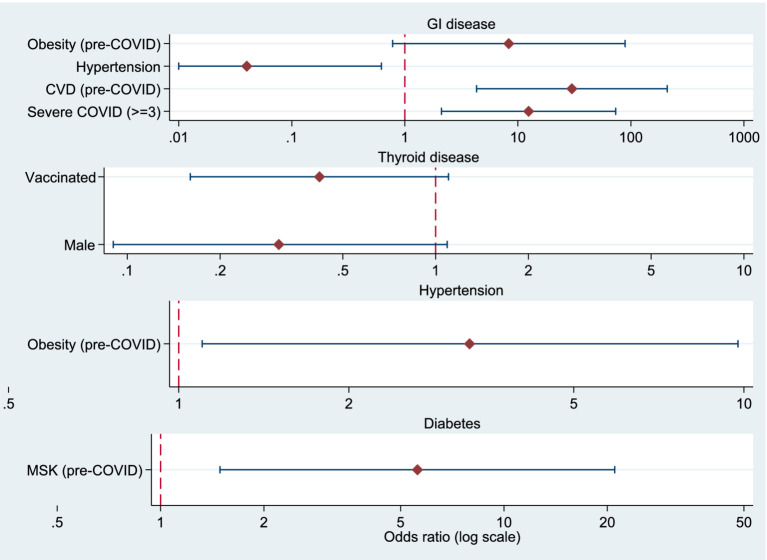
Forest plots showing odds ratios (OR) and 95% confidence intervals for factors associated with post-COVID gastrointestinal diseases, thyroid diseases, arterial hypertension, and diabetes. The vertical dashed line indicates OR = 1. All *x*-axes are presented on a logarithmic scale.

**Figure 3:**
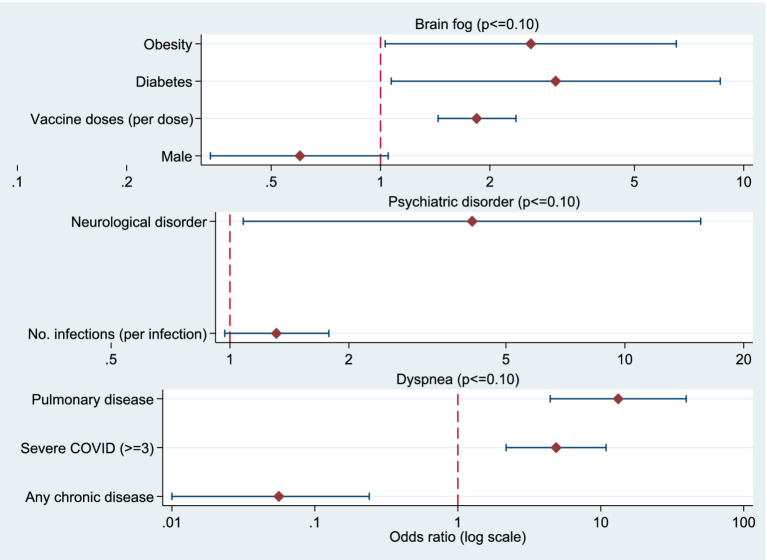
Forest plots showing odds ratios (OR) and 95% confidence intervals for factors associated with brain fog, psychiatric disorders, and dyspnea after COVID-19. The vertical dashed line indicates OR = 1. All *x*-axes are presented on a logarithmic scale.

Odds ratios from the logistic regression analyses are additionally presented as forest plots in the Supplementary material (Figures 1–3).

## 4 Discussion

This study provides valuable insights into the prevalence and risk factors for the development of post-COVID symptoms and diseases in the general population. The findings suggest that while the overall prevalence of post-COVID symptoms is relatively low, they continue to represent a significant public health concern, particularly in terms of their long-term consequences.

The study found that nearly 25% of patients had arterial hypertension before SARS-CoV-2 infection, and almost 13% had other cardiovascular diseases. A total of 4.2% of participants suffered from pulmonary disease before SARS-CoV-2 infection. The analysis showed that chronic pulmonary disease was strongly associated with the severity of SARS-CoV-2 infection. Other studies have demonstrated that conditions such as diabetes, hypertension, coronary heart disease and chronic lung disease significantly increase the risk of severe forms of COVID-19 (5). As these comorbidities can impair immune responses and elevate the risk of complications, their presence further complicates recovery from SARS-CoV-2 infection and contributes to the long-term burden of the disease (15).

The study confirmed that COVID-19 vaccination was a protective factor against severe forms of COVID-19 infection and the development of certain long-term conditions, including musculoskeletal diseases, pulmonary thromboembolism, and pulmonary and thyroid diseases.

In addition to severe COVID-19, our results indicate that male gender, as well as previous cardiovascular and musculoskeletal disease, increase the risk of developing a pulmonary thromboembolic incident. Meta-analyses highlight diabetes and malignant disease as risk factors (16).

Hypertension is known to be associated with post-COVID pulmonary embolism (17), but in our study, arterial hypertension was shown to have a protective effect.

In our study, obesity was shown to be a significant factor associated with both *brain fog* and hypertension. Previous research has similarly shown that obesity can exacerbate the severity of COVID-19 and increase the risk of severe outcomes, including ICU admission and mortality (18). Our results confirm that obesity may be a critical factor in the development of post-COVID cognitive dysfunction, likely due to its impact on metabolic processes and inflammation.

The analysis showed that patients with diabetes, malignancies, chronic kidney and pulmonary disease had higher vaccination rates. This may indicate increased awareness of risk among these patient groups or more proactive health recommendations from healthcare providers.

More than 10% of patients who recovered from SARS-CoV-2 infection developed a chronic illness that is classified as a psychiatric or neurological disorder. Comparing our findings with previous international studies, it is observed that the prevalence of symptoms such as *brain fog* (4.9%) and shortness of breath (2.8%) in this general population sample are consistent with UK population-based studies reporting post-COVID symptom rates of approximately 2–6% (19).

In the systematic review van der Feltz-Cornelis et al. concluded that mental health conditions and *brain fog* both occur in around one in five patients between 3 months and 2 years after SARS-CoV-2 infection and show that the odds of brain fog significantly decreased with increasing vaccination rates (11). An interesting finding of this study is the association between several vaccine doses received and more frequent reporting of *brain fog* symptoms. Although our result may seem contradictory, it is important to emphasize that a causal relationship has not been proven. This association may reflect a higher level of health awareness and willingness of vaccinated subjects to recognize and report symptoms. Another important interpretation of the observed association is that vaccinated groups disproportionately included older adults and individuals with chronic conditions, populations that are inherently more likely to experience and report cognitive symptoms. Vaccine uptake was highest among older participants and those with comorbidities, suggesting that the association with brain fog may reflect underlying demographic and clinical differences rather than a direct effect of vaccination.

Numerous studies indicate a protective effect of vaccination on the occurrence of post-COVID symptoms. Research in Israel has shown that an mRNA vaccine may be associated with a reduced prevalence of reported post-acute SARS-CoV-2 infection symptoms (20).

A study from Norway has also shown that booster vaccination before SARS-CoV-2 infection was associated with a marked reduction in both neurocognitive and cardiorespiratory symptoms persisting for at least 3 months after Omicron infection (21).

Krishna et al. concluded that long COVID has declined markedly in the United Kingdom following vaccine rollout. Although vaccination does not alter the symptom profile, it is associated with reduced symptom severity. These findings suggest that pre-infection immunity lowers long COVID risk, likely through attenuation of acute disease and its sequelae (22).

It is known that cognitive impairment after COVID-19 may be mediated by metabolic and neuroinflammatory processes, which explains the higher frequency of symptoms in people with diabetes and obesity (23). These findings indicate the need for a holistic approach to assessing the post-COVID state, with the family doctor playing a key role in identifying patients at risk.

Research conducted in hospital settings, as expected, revealed a higher prevalence of dyspnea compared to our outpatient sample (24). A meta-analysis on the prevalence of post-COVID breathlessness revealed that 26% of COVID-19 survivors reported the presence of breathlessness symptoms more than 4 weeks post-infection, and 41% of survivors reported reduced physical capacity due to post-COVID breathlessness, as assessed by the MRC/mMRC dyspnoea scale (25). The lower prevalence observed in our sample may be attributed to the milder clinical presentations of infection and the relatively smaller proportion of patients with chronic pulmonary conditions. The results also confirm that dyspnea is one of the most important post-COVID symptoms, especially in people who had a more severe form of acute illness or previous lung disease. Similar associations between the severity of the acute phase and persistent respiratory distress have been found in a review article by Morgan et al. which emphasized that the strongest predictive risk factors for persistent dyspnea included pulmonary comorbidities, the severity of the SARS-CoV-2 infection, female sex, elevated body mass index, pre-existing anxiety and depression, pre-COVID physical limitations, and socioeconomic differences (26). These data further emphasize the importance of systematic monitoring of respiratory function during the recovery period, especially in primary health care, where most patients seek help after discharge from the hospital or after a mild infection at home.

The presence of chronic disease in our results is a protective factor in the development of dyspnea, which can be explained by the fact that people with chronic diseases are usually under more frequent medical supervision and therefore recognize and control respiratory disorders earlier.

The association of neurological and psychiatric disorders after COVID-19 is supported by a growing body of evidence on the neuropsychiatric effects of infection (27). Various theories explaining long COVID, such as direct neuro-invasion, systemic effects of the virus, and neuroimmune dysregulation, have been suggested. Social isolation and associated factors also serve as the link between the SARS-CoV-2 virus, long COVID and its neuropsychiatric manifestations (28). Such patients are particularly vulnerable and require a proactive approach to monitoring and psychosocial support.

From a clinical perspective, family physicians play a crucial role in the early identification and long-term follow-up of patients with post-COVID symptoms (29). As a multisystem disorder, SARS-CoV-2 infection has negative impacts on the physical and mental health of people, as well as their ability to work, so the Croatian Ministry of Health made a guideline for post-COVID rehabilitation. These rehabilitation programs include breathing exercises, physical therapy, psychological support, occupational therapy, education on self-management,etc. in special hospitals for medical rehabilitation, health resorts and thermal spas. The first official Croatian guide for patients recovering from long COVID, which also serves as a guide for health professionals, explains common post-COVID symptoms and how to manage them. The guide offers several benefits for clinical practice: provides a description of common post-COVID symptoms, supports continuity of care and allows healthcare professionals to educate patients on effective self-care strategies (30). Primary care represents the first line of contact for most patients, enabling an integrated approach that encompasses the biomedical, psychological, and social aspects of health. Regular check-ups, assessment of symptoms and coordination of care with other specialists (neurologist, pulmonologist, psychiatrist) can significantly improve patient outcomes and quality of life.

## 5 Limitations

While this study offers important insights into the prevalence and factors influencing post-COVID conditions, several limitations should be considered.

First, the retrospective design of the study may introduce bias, particularly in the recording of diagnoses and the assessment of disease severity. The severity of SARS-CoV-2 infection was determined based on medical documentation, which may not always reflect the full clinical picture, especially in cases with subtle or evolving symptoms. Additionally, as a cross-sectional study, it does not allow for conclusions about causality, particularly regarding the long-term health outcomes of post-COVID conditions. Another limitation is the lack of precise data on the time elapsed between SARS-CoV-2 infection and assessment of post-COVID symptoms. As participants were assessed at varying stages of post-infection recovery, symptom prevalence could not be analyzed by follow-up duration, which should be considered when interpreting the findings.

Fatigue, despite being one of the most common and well-documented post-COVID symptoms, was not specifically assessed in our study. The symptom questionnaire focused primarily on organ-specific symptoms routinely recorded in primary care, and therefore fatigue was not included as a separate variable. This should be considered a limitation when interpreting the prevalence of post-COVID symptoms in our cohort.

The relatively small number of participating practices and the non-random sampling approach limit the generalizability of the findings. Although practices from different regions and settings were included, the sample cannot be considered nationally representative. Potential selection bias cannot be excluded and should be considered when interpreting the results.

## 6 Conclusion

This study provides comprehensive insights into the long-term health effects of SARS-CoV-2 infection in Croatian primary care settings, highlighting the multifaceted nature of post-COVID conditions. Pre-existing chronic diseases, older age, and obesity emerged as significant risk factors for more severe acute COVID-19 and the subsequent development of long-term complications, including neurological, psychiatric, cardiovascular, pulmonary, and metabolic sequelae. Notably, COVID-19 vaccination demonstrated consistent protective effects against severe disease and certain post-COVID conditions, underscoring its critical role in mitigating long-term health impacts.

Although the overall prevalence of post-COVID symptoms in the general population was lower than in hospitalized patients, cognitive dysfunction, dyspnea, and other persistent symptoms remain substantial public health concerns. The study also revealed nuanced associations, such as the increased reporting of brain fog among individuals with higher vaccination doses, likely reflecting heightened health awareness and underlying demographic and clinical factors rather than a causal effect. These findings reinforce the complex interplay between pre-existing conditions, vaccination, and post-COVID outcomes, particularly in populations with metabolic and chronic health conditions where inflammatory and neuroimmune pathways may drive symptom persistence.

Family physicians are uniquely positioned to provide comprehensive, patient-centered care that integrates biomedical, psychological, and social aspects. Early identification of at-risk patients, systematic monitoring of symptoms, and timely interventions can mitigate complications, improve functional outcomes, and reduce the long-term burden of post-COVID conditions on both patients and healthcare systems. This study emphasizes the importance of vigilant, targeted preventive strategies and the central role of primary care in managing post-COVID health, especially in patients with chronic comorbidities.

Despite the valuable insights provided, the study’s retrospective design, lack of standardized follow-up data, absence of fatigue assessment, and non-random sample limit the generalizability of the findings. Future prospective, longitudinal studies with representative populations are essential to validate these results, clarify symptom trajectories, and further elucidate the complex relationships between pre-existing conditions, vaccination, and long-term post-COVID sequelae.

In summary, sustained vigilance, targeted immunization strategies, integrated primary care, and patient-centered follow-up are critical for addressing the enduring health consequences of SARS-CoV-2 infection and safeguarding population health.

## Data Availability

The raw data supporting the conclusions of this article will be made available by the authors, without undue reservation.
